# Characterization of the complete chloroplast genome of a Chinese Endangered species *Cymbidium mastersii*

**DOI:** 10.1080/23802359.2020.1851152

**Published:** 2021-03-15

**Authors:** Qing Ling, Longjie Cheng, Zhilin Li, Yiran Zhao, Yuying Wang

**Affiliations:** a College of Horticulture and Landscape, Yunnan Agricultural University, Kunming, China; b Yunnan Vocational and Technical College of Agriculture, Kunming, China

**Keywords:** Cymbidium mastersii, chloroplast genome, endangered species, phylogenetic analysis

## Abstract

*Cymbidium mastersii* is an endangered species belonging to the first ranking in protection category in China with important ornamental value and breeding value. This study used Illumina high-throughput sequencing technologies for *C. mastersii* of chloroplast genome sequencing. The genome features of *C. mastersii* and the phylogenetic relationships were reported and established. The complete chloroplast genome is 156,317 bp in length, consisting of a pair of inverse duplication regions 26,544 bp, a large single-copy region 85,360 bp and a small single-copy region 17,869 bp. The entire genome contains 73 mRNA genes, 30 tRNA genes and 4 rRNA genes. The phylogenetic tree of 23 Orchidaceae species revealed *C. mastersii* is more closely related to *Cymbidium eburneum*.

*Cymbidium mastersii* (Orchidaceae) is a shrub that is one of the endemic species in Yunnan province, China. The wild *C. mastersii* grows on trees or rocks in forests at elevations of 1600–1800 m (Liu et al. 2009). It has been listed as a Class I protected plant in the China biodiversity Red List and in the China Rare and Endangered Plants List (http://www.iplant.cn/rep/protlist). *Cymbidium mastersii* is an excellent garden plant with white flowers that have extraordinary aromas of almond. The length of inflorescence is 25–45 cm with 2–5 or more flowers, and the flowering period ranges from September to December (Guo et al. 2012). Due to its unique scent, beautiful flower shape and colors, the *C. mastersii* is used as a material for potted flowers and an important germplasm resource for breeding.

The complete chloroplast genome sequences of *C. mastersii* was obtained (GenBank Accession No. MT576627). The genome sequences and features are significant to study the phylogenetic relationship of *C. mastersii* and help speed up the in-depth research of chloroplast. Besides, it plays an essential role in the diversity research of genetic resources of this plant. Specimen of *C. mastersii* were gathered from the Flower Research Institute of College of Horticulture and Landscape, Yunnan Agricultural University, Kunming, Yunnan Province, China (25°07′43″ N, 102°44′54″ E), and specimens were deposited in the Herbarium of Kunming Institute of Botany of CAS (specimen code: CY002). The improved CTAB method (Doyle and Doyle 1987) was used to extract the entire chloroplast DNA of *C. mastersii* from fresh mesophyll tissue.

Sequencing the DNA was performed using the Illumina NovaSeq in GENOSEQ Technologies Limited Company (Wuhan, China). Namely, the raw reads and clean reads were obtained and then were assembled by NovoPlasty (Dierckxsens et al. 2017). The assembled contigs were compared with the chloroplast genomes of the closely related species through the use of blastn (version: BLAST 2.2.30+; parameter: -evalue 1e–5). Then the contigs were checked, selected, adjusted to get the final data. The chloroplast genome was annotated and mapped using GeSeq (Tillich et al. 2017).

The length of complete chloroplast genome of *C. mastersii* is 156,317 bp. The genome presented a characteristic quadripartite circular structure which included one pair of inverted repeat regions (IRs, 26,544 bp), one large single-copy region (LSC, 85,360 bp) and one small single-copy region (SSC, 17,869 bp). Besides, the complete genome contains 73 mRNA genes, 30 tRNA genes and 4 rRNA genes. The overall GC content of *C. mastersii* chloroplast genome is 36.74%. Moreover, the GC content of IR regions (43.09%) is higher than the LSC region (34.29%) and the SSC region (29.61%).

To study the phylogenetic relationship of *C. mastersii*, a phylogenetic tree was constructed by using 20 complete chloroplast genomes of *Cymbidium* species and three *Orchidaceae* species were selected as an outgroup. All the sequences were downloaded from NCBI GenBank and then aligned using the online program MAFFT version 7. MEGA version 7.0 was used to build the maximum-likelihood phylogenetic tree with 1000 rapid bootstrap replicates (Kumar et al. 2016). The phylogenetic tree analysis indicated that *Cymbidium mastersii* was closely related to *Cymbidium eburneum* (Figure 1).

**Figure 1.**
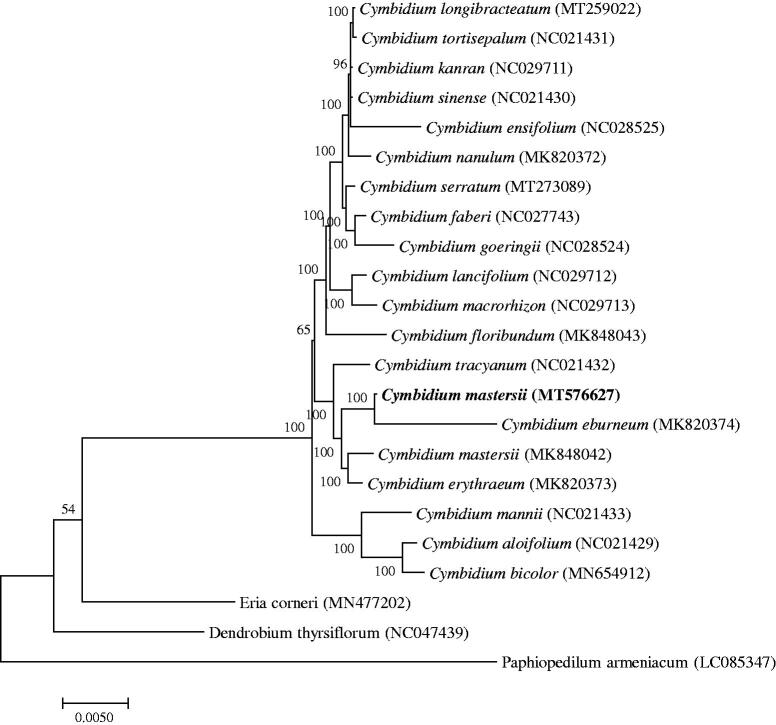
A Phylogenetic tree based on 23 complete chloroplast genome sequences of Orchidaceae species using the maximum likelihood (ML) analysis by MEGA version 7.0. Bootstrap support values are indicated in each node.

The complete chloroplast genome of *C. mastersii* (MT576627) in our study and a published complete genome of *C. mastersii* (MK848042) differ from each other. The specimens of *C. mastersii* (MK848042) were gathered from Shenzhen, China in 2019, the complete chloroplast genome was 155,362 bp in length and contained 80 protein-coding genes, 23 tRNAs, and 4 rRNAs (Fang et al. 2019). In our research, the *C. mastersii* (MT576627) was collected from the wild resource of Yunnan province in 2020, and the complete chloroplast genome was 156,317 bp in length and contained 73 protein-coding genes, 30 tRNA genes and 4 rRNA genes. Therefore, both of *C. mastersii* (MT576627) and *C. mastersii* (MK848042) are different in sample location,year,the length and number of protein-coding, tRNA and rRNA of complete chloroplast genome.

## Data Availability

The data that support the findings of this study are openly available in GenBank of NCBI at https://www.ncbi.nlm.nih.gov, reference number MT576627.
